# Amelioration of Enterotoxigenic *Escherichia coli*-Induced Intestinal Barrier Disruption by Low-Molecular-Weight Chitosan in Weaned Pigs is Related to Suppressed Intestinal Inflammation and Apoptosis

**DOI:** 10.3390/ijms20143485

**Published:** 2019-07-16

**Authors:** Jin Wan, Jiao Zhang, Guozhong Wu, Daiwen Chen, Bing Yu, Zhiqing Huang, Yuheng Luo, Ping Zheng, Junqiu Luo, Xiangbing Mao, Jie Yu, Jun He

**Affiliations:** 1 Institute of Animal Nutrition, Sichuan Agricultural University, Chengdu 611130, China; 2 Shanghai Institute of Applied Physics, Chinese Academy of Sciences, Shanghai 201800, China

**Keywords:** low-molecular-weight chitosan, *Escherichia coli*, intestinal integrity, inflammatory responses, cell apoptosis, weaned pigs

## Abstract

Enterotoxigenic *Escherichia coli* (ETEC) infection destroys the intestinal barrier integrity, in turn, disrupting intestinal homoeostasis. Low-molecular-weight chitosan (LMWC) is a water-soluble chitosan derivative with versatile biological properties. Herein, we examined whether LMWC could relieve ETEC-induced intestinal barrier damage in weaned pigs. Twenty-four weaned pigs were allotted to three treatments: (1) non-infected control; (2) ETEC-infected control; and (3) ETEC infection + LMWC supplementation (100 mg/kg). On day 12, pigs in the infected groups were administered 100 mL of ETEC at 2.6 × 10^9^ colony-forming units/mL to induce intestinal barrier injury. Three days later, serum samples were obtained from all pigs, which were then slaughtered to collect intestinal samples. We evidenced that LMWC not only increased (*P* < 0.05) the occludin protein abundance but also decreased (*P* < 0.05) the interleukin-6, tumour necrosis factor-α and mast cell tryptase contents, and the apoptotic epithelial cell percentages, in the small intestine of ETEC-infected pigs. Furthermore, LMWC down-regulated (*P* < 0.05) the small intestinal expression levels of critical inflammatory- and apoptotic-related genes, such as Toll-like receptor 4 (*TLR4*) and tumour necrosis factor receptor 1 (*TNFR1*), as well as the intra-nuclear nuclear factor-κB (NF-κB) p65 protein abundance, in the ETEC-infected pigs. Our study indicated a protective effect of LMWC on ETEC-triggered intestinal barrier disruption in weaned pigs, which involves the repression of intestinal inflammatory responses via blocking the TLR4/NF-κB signalling pathway and the depression of epithelial cell death via TNFR1-dependent apoptosis.

## 1. Introduction 

The small intestine not only serves as a major digestive and absorptive organ for nutrients [1] but also functions as a protective barrier to prevent the penetration of luminal antigens, microorganisms and toxins from entering the internal environment [2,3]. However, enteric pathogens, such as enterotoxigenic *Escherichia coli* (ETEC), colonise the small intestine and release enterotoxins that induce severe diarrhoea and impair the intestinal barrier integrity [4,5,6]. To date, the pathogenesis of intestinal injury caused by ETEC has not been fully clarified. Previous studies have shown that the over-production of pro-inflammatory mediators upon ETEC infection is an inducing factor that leads to disruption of the intestinal barrier integrity [7,8]. Moreover, ETEC infection has been reported to promote apoptosis in intestinal epithelial cells, further compromising the intestinal barrier integrity [9,10]. Therefore, suppressing the production of intestinal pro-inflammatory mediators and inhibiting epithelial cell apoptosis could attenuate ETEC-induced disruption of the intestinal barrier.

Chitosan is the product of deacetylation of chitin, which is derived from the exoskeletons of arthropods and fungi cell walls, and is the second most abundant natural biomass resource [11]. Despite its biocompatibility, biodegradability and non-toxicity, chitosan is water-insoluble, which has greatly limited its practical applications [12,13]. This issue has been resolved by the depolymerisation of chitosan into a water-soluble derivative termed low-molecular-weight-chitosan (LMWC) [14]. LMWC possesses a wide range of biological activities, including anti-inflammatory and immunoregulatory actions [15], making it an attractive candidate for use in food, agricultural and medical industries. A recent study revealed that LMWC supplementation was beneficial to maintaining the intestinal barrier function in pigs after weaning [16]. Nevertheless, no prior information is available concerning the influences of LMWC supplementation on intestinal repair in weaned pigs challenged with ETEC. Therefore, further investigations focused on this theme would be of great interest.

Accordingly, the purpose of this study was to elaborate on the efficacy of LMWC supplementation against ETEC-induced intestinal barrier injury and decipher the underlying mechanisms. Our results may provide theoretical support for the future application of LMWC in the prevention and treatment of ETEC-induced intestinal injury in both animals and humans.

## 2. Results

### 2.1. Growth Performance

**Figure 1:**
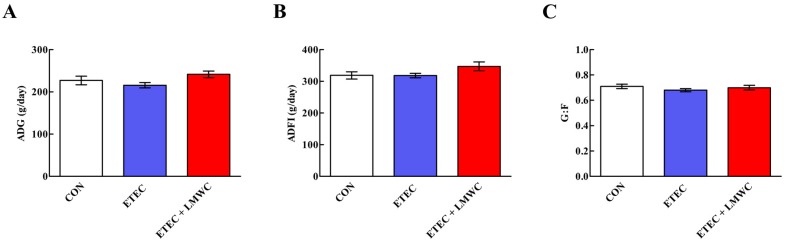
Growth performance of weaned pigs among the three groups (Days 1–14). (**A**) ADG, average daily gain. (**B**) ADFI, average daily feed intake. (**C**) G:F, gain-to-feed ratio. Values are means (*n* = 8 pigs/treatment), with standard errors represented by vertical bars. CON: non-infected control (pigs received the basal diet and infused with sterilised Luria–Bertani culture); ETEC: ETEC-infected control (pigs received the basal diet and infused with enterotoxigenic *Escherichia coli*); ETEC + LMWC: pigs received the basal diet supplemented with 100 mg/kg low-molecular-weight chitosan and infused with enterotoxigenic *Escherichia coli*.

Throughout the 2-week trial, there were no variations (*P* > 0.05) in the average daily gain (ADG), average daily feed intake (ADFI) and gain-to-feed ratio (G:F) among the three groups (Figure 1).

### 2.2. Serum Indices

**Figure 2:**
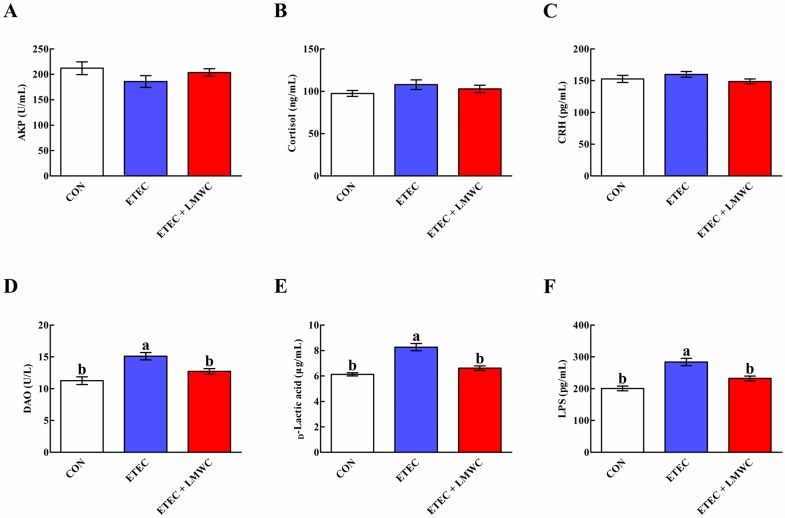
Effects of low-molecular-weight chitosan on the serum indices in weaned pigs challenged with enterotoxigenic *Escherichia coli*. (**A**) AKP, alkaline phosphatase. (**B**) Cortisol. (**C**) CRH, corticotropin-releasing hormone. (**D**) DAO, diamine oxidase. (**E**) d-Lactic acid. (**F**) LPS, lipopolysaccharide. ^a, b^ Means with different letters on vertical bars indicate significant differences (*P* < 0.05). Values are means (*n* = 8 pigs/treatment), with standard errors represented by vertical bars. CON: non-infected control (pigs received the basal diet and infused with sterilised Luria–Bertani culture); ETEC: ETEC-infected control (pigs received the basal diet and infused with enterotoxigenic *Escherichia coli*); ETEC + LMWC: pigs received the basal diet supplemented with 100 mg/kg low-molecular-weight chitosan and infused with enterotoxigenic *Escherichia coli*.

As presented in Figure 2, serum d-lactic acid and lipopolysaccharide (LPS) contents, as well as diamine oxidase (DAO) activity, were increased (*P* < 0.05) after ETEC infection, compared with the non-infected control group, while these phenomena were reversed (*P* < 0.05) by LMWC supplementation. Neither ETEC nor LMWC affected (*P* > 0.05) the serum alkaline phosphatase (AKP) activity and cortisol and corticotropin-releasing hormone (CRH) concentrations.

### 2.3. Intestinal Integrity

**Figure 3:**
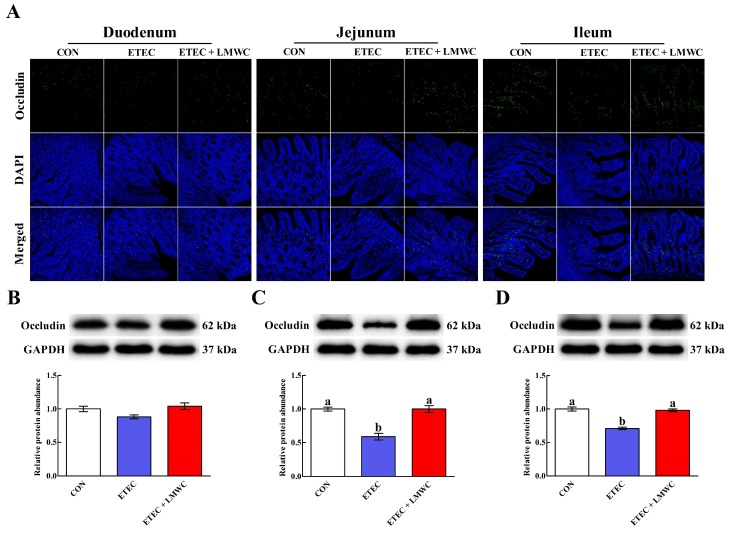
Effects of low-molecular-weight chitosan on the occludin distribution and expression in the small intestine of weaned pigs challenged with enterotoxigenic *Escherichia coli*. (**A**) Duodenal, jejunal and ileal occludin distribution (images were acquired by laser scanning confocal microscopy; magnification ×200). (**B**–**D**) Duodenal, jejunal and ileal occludin protein abundance, respectively. ^a, b^ Means with different letters on vertical bars indicate significant differences (*P* < 0.05). Values are means (*n* = 8 pigs/treatment), with standard errors represented by vertical bars. CON: non-infected control (pigs received the basal diet and infused with sterilised Luria–Bertani culture); ETEC: ETEC-infected control (pigs received the basal diet and infused with enterotoxigenic *Escherichia coli*); ETEC + LMWC: pigs received the basal diet supplemented with 100 mg/kg low-molecular-weight chitosan and infused with enterotoxigenic *Escherichia coli*. DAPI, 4′,6-diamidino-2-phenylindole. GAPDH, glyceraldehyde-3-phosphate dehydrogenase.

The immunofluorescence assay for occludin showed that its expression and localisation in the apical intercellular region of the small intestinal epithelium was influenced by either ETEC infection or LMWC supplementation (Figure 3). This effect was verified by western blot analysis, which illustrated that ETEC infection decreased (*P* < 0.05) the jejunal and ileal occludin protein abundances. Interestingly, dietary supplementation with LMWC attenuated (*P* < 0.05) the reduced jejunal and ileal occludin protein abundances caused by ETEC invasion.

### 2.4. Intestinal Cytokine Content

**Figure 4:**
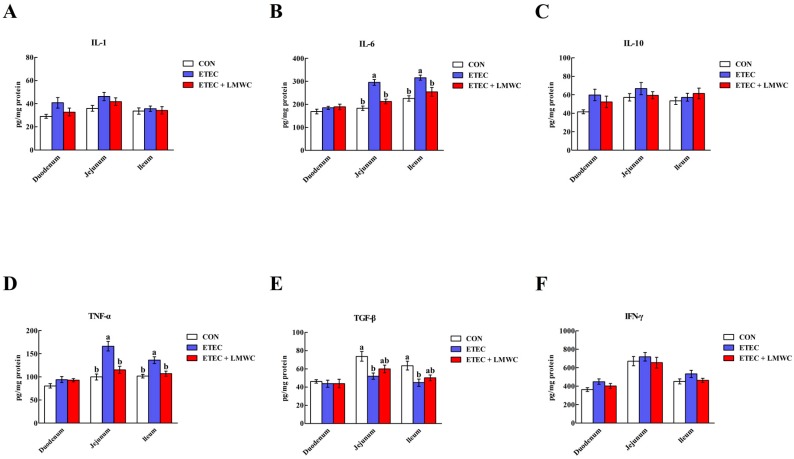
Effects of low-molecular-weight chitosan on the intestinal cytokine contents of weaned pigs challenged with enterotoxigenic *Escherichia coli*. (**A**) IL-1, interleukin-1. (**B**) IL-6, interleukin-6. (**C**) IL-10, interleukin-10. (**D**) TNF-α, tumour necrosis factor-α. (**E**) TGF-β, transforming growth factor-β. (**F**) IFN-γ, interferon-γ. ^a, b^ Means with different letters on vertical bars indicate significant differences (*P* < 0.05). Values are means (*n* = 8 pigs/treatment), with standard errors represented by vertical bars. CON: non-infected control (pigs received the basal diet and infused with sterilised Luria–Bertani culture); ETEC: ETEC-infected control (pigs received the basal diet and infused with enterotoxigenic *Escherichia coli*); ETEC + LMWC: pigs received the basal diet supplemented with 100 mg/kg low-molecular-weight chitosan and infused with enterotoxigenic *Escherichia coli*.

From Figure 4, it was possible to observe that the elevated (*P* < 0.05) interleukin-6 (IL-6) and tumour necrosis factor-α (TNF-α) contents and reduced (*P* < 0.05) transforming growth factor-β (TGF-β) content in the jejunal and ileal mucosae were induced by ETEC challenge. However, LMWC supplementation decreased (*P* < 0.05) the IL-6 and TNF-α contents in the jejunal and ileal mucosae of ETEC-challenged pigs. Moreover, duodenal, jejunal and ileal IL-1, IL-10 and interferon-γ (IFN-γ) contents did not differ (*P* > 0.05) among the three treatments.

### 2.5. Intestinal Mast Cell Protease Content

**Figure 5:**
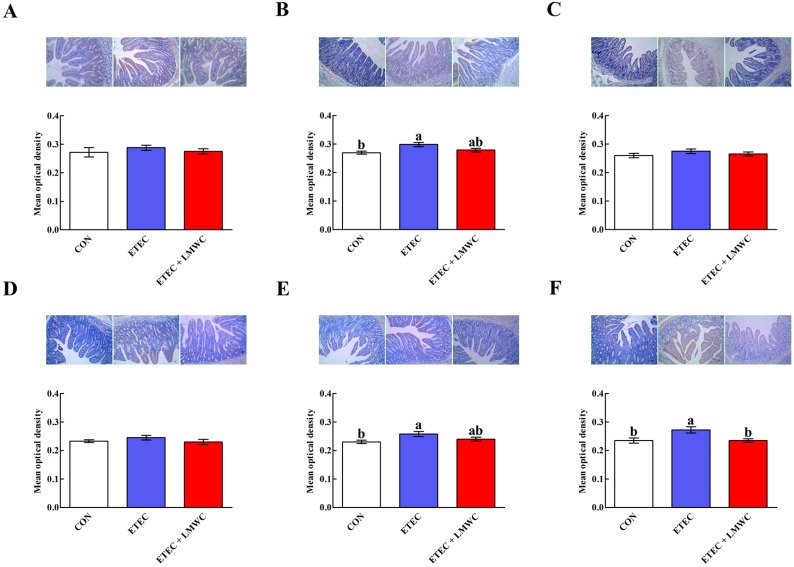
Effects of low-molecular-weight chitosan on the mast cell chymase and tryptase contents in the small intestine of weaned pigs challenged with enterotoxigenic *Escherichia coli* (images were acquired by digital microscopy; magnification ×200). (**A**–**C**) Duodenal, jejunal and ileal mast cell chymase contents, respectively. (**D**–**F**) Duodenal, jejunal and ileal mast cell tryptase contents, respectively. ^a, b^ Means with different letters on vertical bars indicate significant differences (*P* < 0.05). Values are means (*n* = 8 pigs/treatment), with standard errors represented by vertical bars. CON: non-infected control (pigs received the basal diet and infused with sterilised Luria–Bertani culture); ETEC: ETEC-infected control (pigs received the basal diet and infused with enterotoxigenic *Escherichia coli*); ETEC + LMWC: pigs received the basal diet supplemented with 100 mg/kg low-molecular-weight chitosan and infused with enterotoxigenic *Escherichia coli*. Scale bar is 40 μm.

After challenge with ETEC, jejunal mast cell chymase and tryptase contents, and the ileal mast cell tryptase content were increased (*P* < 0.05; Figure 5). However, dietary incorporation of LMWC decreased (*P* < 0.05) the ileal mast cell tryptase content in the ETEC-infected pigs. Neither ETEC nor LMWC affected (*P* > 0.05) the duodenal mast cell chymase and tryptase contents.

### 2.6. Intestinal Toll-like Receptor 4 (TLR4) Signalling Pathway-Related Gene Expression

**Figure 6:**
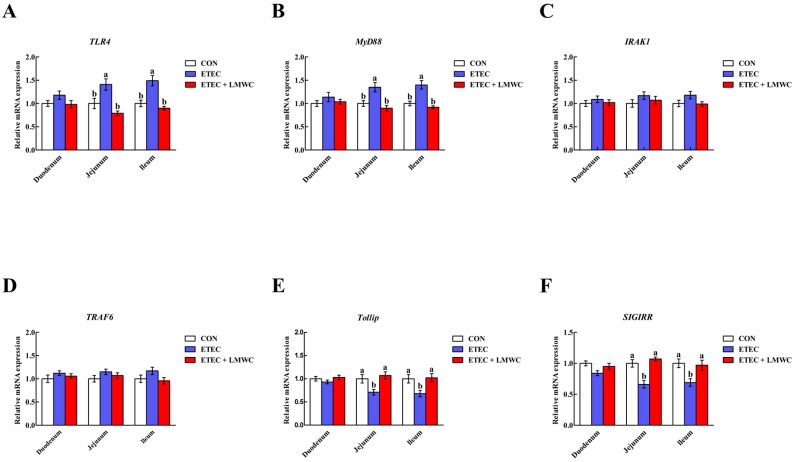
Effects of low-molecular-weight chitosan on the mRNA levels of *TLR4* (**A**), *MyD88* (**B**), *IRAK1* (**C**), *TRAF6* (**D**), *Tollip* (**E**) and *SIGIRR* (**F**) in the small intestine of weaned pigs challenged with enterotoxigenic *Escherichia coli*. (**A**) *TLR4*, Toll-like receptor 4. (**B**) *MyD88*, myeloid differentiation factor 88. (**C**) *IRAK1*, interleukin-1 receptor-associated kinase 1. (**D**) *TRAF6*, tumour necrosis factor receptor-associated factor 6. (**E**) *Tollip*, Toll-interacting protein. (**F**) *SIGIRR*, single immunoglobulin interleukin-1 receptor-related molecule. ^a, b^ Means with different letters on vertical bars indicate significant differences (*P* < 0.05). Values are means (*n* = 8 pigs/treatment), with standard errors represented by vertical bars. CON: non-infected control (pigs received the basal diet and infused with sterilised Luria–Bertani culture); ETEC: ETEC-infected control (pigs received the basal diet and infused with enterotoxigenic *Escherichia coli*); ETEC + LMWC: pigs received the basal diet supplemented with 100 mg/kg low-molecular-weight chitosan and infused with enterotoxigenic *Escherichia coli*.

The ETEC group exhibited higher (*P* < 0.05) mRNA levels of *TLR4* and myeloid differentiation factor 88 (*MyD88*), and lower mRNA levels of Toll-interacting protein (*Tollip*) and single immunoglobulin interleukin-1 receptor-related molecule (*SIGIRR*) in the jejunal and ileal mucosae, compared with the non-infected control group (Figure 6). Conversely, lower (*P* < 0.05) mRNA levels of *TLR4* and *MyD88*, and higher (*P* < 0.05) mRNA levels of *Tollip* and *SIGIRR* in the jejunal and ileal mucosae, were observed in the ETEC + LMWC group than those in the ETEC group. In addition, the mRNA levels of interleukin-1 receptor-associated kinase 1 (*IRAK1*) and tumour necrosis factor receptor-associated factor 6 (*TRAF6*) in the three intestinal mucosae were not altered (*P* > 0.05) by either ETEC or LMWC.

### 2.7. Intestinal Nuclear Factor-κB (NF-κB) p65 Protein Abundance

**Figure 7:**
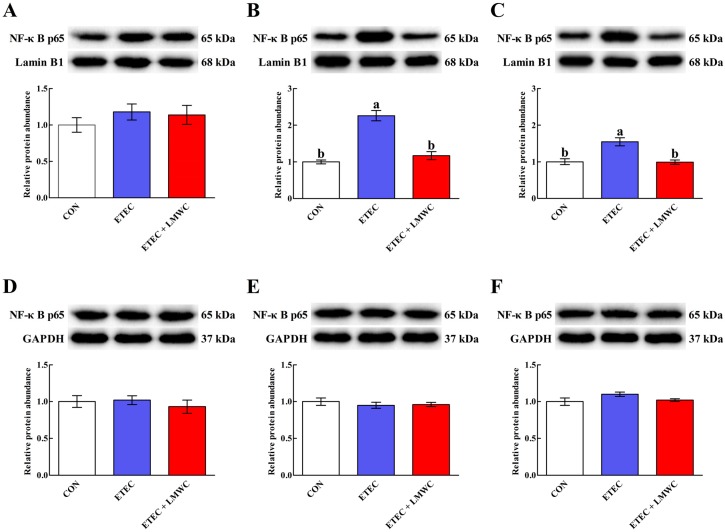
Effects of low-molecular-weight chitosan on the nuclear and cytoplasmic NF-κB p65 protein abundances in the small intestine of weaned pigs challenged with enterotoxigenic *Escherichia coli*. (**A**–**C**) Duodenal, jejunal and ileal nuclear NF-κB p65 protein abundances, respectively. (**D**–**F**) Duodenal, jejunal and ileal cytoplasmic NF-κB p65 protein abundances, respectively. ^a, b^ Means with different letters on vertical bars indicate significant differences (*P* < 0.05). Values are means (*n* = 8 pigs/treatment), with standard errors represented by vertical bars. CON: non-infected control (pigs received the basal diet and infused with sterilised Luria–Bertani culture); ETEC: ETEC-infected control (pigs received the basal diet and infused with enterotoxigenic *Escherichia coli*); ETEC + LMWC: pigs received the basal diet supplemented with 100 mg/kg low-molecular-weight chitosan and infused with enterotoxigenic *Escherichia coli*. NF-κB p65, nuclear factor-κB p65. GAPDH, glyceraldehyde-3-phosphate dehydrogenase.

The ETEC infection only up-regulated (*P* < 0.05) the jejunal and ileal mucosal nuclear NF-κB p65 protein abundances without modifying (*P* > 0.05) the cytoplasmic NF-κB p65 protein abundances in the duodenal, jejunal and ileal mucosae (Figure 7). However, nuclear NF-κB p65 protein abundances declined (*P* < 0.05) in the jejunal and ileal mucosae of ETEC-infected pigs following LMWC ingestion.

### 2.8. Enterocyte Apoptosis Percentage

**Figure 8:**
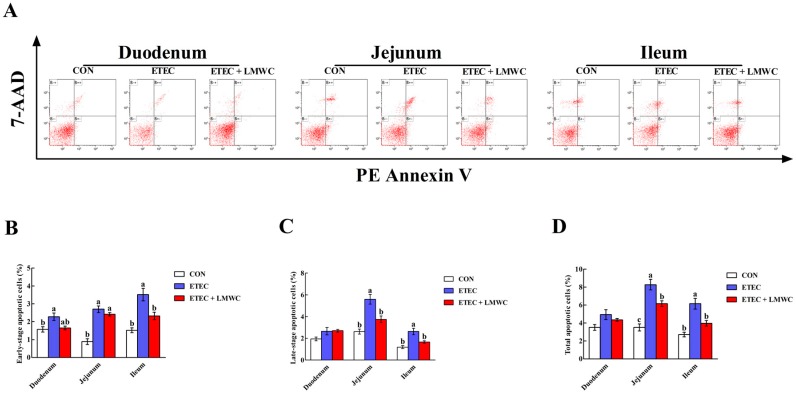
Effects of low-molecular-weight chitosan on the epithelial cell apoptosis in the small intestine of weaned pigs challenged with enterotoxigenic *Escherichia coli*. (**A**) Representative scattergram of apoptotic epithelial cells in the small intestine of weaned pigs among the three groups. (**B**) Early-stage apoptotic cells. (**C**) Late-stage apoptotic cells. (**D**) Total apoptotic cells. ^a–c^ Means with different letters on vertical bars indicate significant differences (*P* < 0.05). Values are means (*n* = 8 pigs/treatment), with standard errors represented by vertical bars. CON: non-infected control (pigs received the basal diet and infused with sterilised Luria–Bertani culture); ETEC: ETEC-infected control (pigs received the basal diet and infused with enterotoxigenic *Escherichia coli*); ETEC + LMWC: pigs received the basal diet supplemented with 100 mg/kg low-molecular-weight chitosan and infused with enterotoxigenic *Escherichia coli*. Cells that stain negative for PE Annexin V and positive for 7-AAD are necrotic cells (B−+); cells that stain positive for both PE Annexin V and 7-AAD are in the late-stage of apoptosis (B++); cells that stain negative for both PE Annexin V and 7-AAD are normal cells (B−−); cells that stain positive for PE Annexin V and negative for 7-AAD are undergoing the early-stage of apoptosis (B+−). 7-AAD, 7-aminoactinomycin D.

The effects of LMWC on the enterocyte apoptosis in weaned pigs challenged with ETEC are shown in Figure 8. The ETEC enhanced (*P* < 0.05) the percentages of early-stage, late-stage and total apoptotic cells in the duodenal, jejunal and ileal epithelia, and the late-stage and total apoptotic cells in the jejunal and ileal epithelia. Importantly, LMWC abrogated (*P* < 0.05) the percentages of early-stage, late-stage and total apoptotic cells in the jejunal and ileal epithelia that were enhanced in ETEC-challenged pigs.

### 2.9. Enterocyte Apoptosis-Related Gene Expression

**Figure 9:**
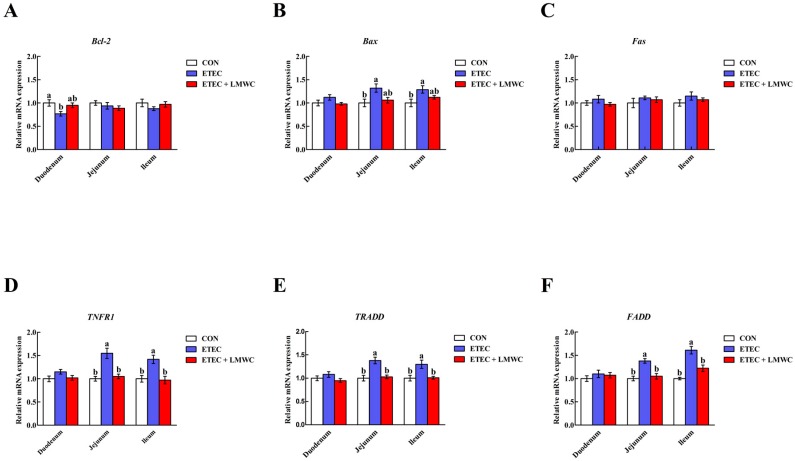
Effects of low-molecular-weight chitosan on the mRNA levels of *Bcl-2* (**A**), *Bax* (**B**), *Fas* (**C**), *TNFR1* (**D**), *TRADD* (**E**) and *FADD* (**F**) in the small intestine of weaned pigs challenged with enterotoxigenic *Escherichia coli*. (**A**) *Bcl-2*, B-cell lymphoma-2. (**B**) *Bax*, B-cell lymphoma-2-associated X protein. (**C**) *Fas*. (**D**) *TNFR1*, tumour necrosis factor receptor 1. (**E**) *TRADD*, tumour necrosis factor receptor-associated death domain. (**F**) *FADD*, Fas-associated death domain. ^a, b^ Means with different letters on vertical bars indicate significant differences (*P* < 0.05). Values are means (*n* = 8 pigs/treatment), with standard errors represented by vertical bars. CON: non-infected control (pigs received the basal diet and infused with sterilised Luria–Bertani culture); ETEC: ETEC-infected control (pigs received the basal diet and infused with enterotoxigenic *Escherichia coli*); ETEC + LMWC: pigs received the basal diet supplemented with 100 mg/kg low-molecular-weight chitosan and infused with enterotoxigenic *Escherichia coli*.

Compared with the non-infected control group, the pigs infected with ETEC had higher (*P* < 0.05) jejunal and ileal mucosal B-cell lymphoma-2-associated X protein (*Bax*), tumour necrosis factor receptor 1 (*TNFR1*), TNFR-associated death domain (*TRADD*) and Fas-associated death domain (*FADD*) mRNA levels, and a lower (*P* < 0.05) duodenal mucosal B-cell lymphoma-2 (*Bcl*-*2*) mRNA level (Figure 9). However, dietary LMWC inclusion limited (*P* < 0.05) the ETEC-induced up-regulation of *TNFR1*, *TRADD* and *FADD* mRNA levels in the jejunal and ileal mucosae. Furthermore, duodenal, jejunal and ileal mucosal *Fas* mRNA levels were unaffected (*P* > 0.05) by either ETEC or LMWC.

### 2.10. Intestinal Cleaved Cysteinyl Aspartate-Specific Protease (Caspase) Contents

**Figure 10:**
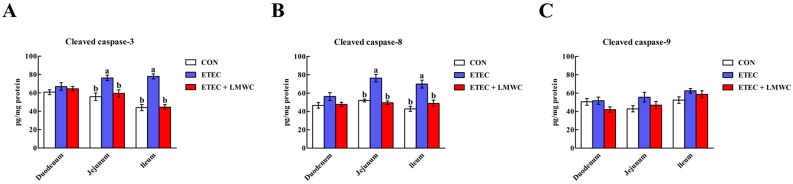
Effects of low-molecular-weight chitosan on the cleaved caspase contents in the small intestine of weaned pigs challenged with enterotoxigenic *Escherichia coli*. (**A**) Cleaved caspase-3, cleaved cysteinyl aspartate-specific protease-3. (**B**) Cleaved caspase-8, cleaved cysteinyl aspartate-specific protease-8. (**C**) Cleaved caspase-9, cleaved cysteinyl aspartate-specific protease-9. ^a, b^ Means with different letters on vertical bars indicate significant differences (*P* < 0.05). Values are means (*n* = 8 pigs/treatment), with standard errors represented by vertical bars. CON: non-infected control (pigs received the basal diet and infused with sterilised Luria–Bertani culture); ETEC: ETEC-infected control (pigs received the basal diet and infused with enterotoxigenic *Escherichia coli*); ETEC + LMWC: pigs received the basal diet supplemented with 100 mg/kg low-molecular-weight chitosan and infused with enterotoxigenic *Escherichia coli*.

According to Figure 10, the cleaved caspase-3 and -8 contents in the jejunal and ileal mucosae were enhanced (*P* < 0.05) post-ETEC challenge, whereas, LMWC supplementation attenuated (*P* < 0.05) these ETEC-induced effects. No alterations (*P* > 0.05) of the cleaved caspase-9 content in the duodenal, jejunal and ileal mucosae were detected among the treatment groups.

## 3. Discussion

The intestinal barrier integrity is maintained by cell–cell cohesion via tight junctions [17]. The tight junction is a multifunctional complex that forms a seal between adjacent epithelial cells near the apical surface [18,19]. These highly dynamic complexes seal the paracellular space between epithelial cells, thereby preventing the paracellular diffusion of microorganisms and other antigens across the epithelium. However, infection of weaned pigs with ETEC is commonly accompanied by disruption of the intestinal barrier integrity, due to tight junction alterations [20,21]. At present, we confirmed that occludin, an integral membrane protein of the epithelial tight junction assembly that contributes to maintaining the intestinal barrier integrity [22,23], was less expressed and localised in the apical intercellular region of the jejunal and ileal epithelia in the ETEC group than the non-infected control group. Importantly, LMWC supplementation increased the jejunal and ileal occludin protein abundances in weaned pigs challenged with ETEC, indicating that LMWC could protect the weaned pigs against ETEC-induced intestinal barrier impairment. In addition, DAO and d-lactic acid serve as indicators of intestinal integrity, as they are normally present in minute amounts in the circulation. Increased serum DAO activity and d-lactic acid concentration reflect changes in intestinal permeability, suggesting that the intestinal barrier function has been damaged [24,25]. In this study, we noticed that LMWC intervention counteracted serum d-lactic acid concentration and DAO activity increments engendered by ETEC in weaned pigs, further verifying the view mentioned above.

Cytokines exert momentous influences on the inflammatory responses and participate in the regulation of intestinal barrier integrity [26]. An imbalance of pro- and anti-inflammatory cytokines has been associated with ETEC-induced intestinal barrier disruption [27,28]. Consistently, the present study showed that ETEC infection increased the IL-6 and TNF-α contents, and decreased the TGF-β content, in the jejunal and ileal mucosae, concurrent with an elevated disruption of the jejunal and ileal barrier. Nonetheless, LMWC supplementation attenuated the elevated jejunal and ileal mucosal IL-6 and TNF-α contents resulting from ETEC infection in weaned pigs. As such, LMWC might prevent the inflammatory responses incurred by ETEC in weaned pigs. In addition, there is evidence that points to a role for mast cells in the modulation of inflammatory responses and intestinal barrier integrity [29,30,31]. These cells contain large amounts of preformed compounds, commonly referred to as mast cell inflammatory mediators, such as chymase and tryptase [32]. Once activated, mast cells release these mediators that inflict intestinal barrier dysfunction and increase intestinal permeability [33,34]. The present study showed that mast cell degranulation engendered by ETEC was partially prevented by LMWC, as LMWC decreased the ileal mast cell tryptase content in ETEC-infected pigs. Taken together, dietary supplementation with LMWC has beneficial effects in mitigating ETEC-induced intestinal inflammation and likely contributed to improving the intestinal integrity in weaned pigs infected with ETEC.

Intestinal barrier damage caused by inflammation can be mediated by the inflammatory responses triggered when pathogens and TLRs interact [35,36]. Among the TLRs, TLR4 is the best-studied member and responds primarily to LPS, a component derived from the outer membrane of Gram-negative bacteria, which induces down-stream signalling events that lead to NF-κB activation, in turn, motivating inflammatory responses through enhancing the expression of pro-inflammatory cytokines [37]. In the present study, coincident with the changes in pro-inflammatory cytokine levels, we observed that LMWC down-regulated the jejunal and ileal mucosal *TLR4* mRNA levels and the mRNA level of its down-stream signal *MyD88*, in ETEC-challenged pigs. Aside from these effects, the ETEC-induced increased nuclear NF-κB p65 protein abundances in the jejunal and ileal mucosae of weaned pigs were inhibited, to a certain extent, by LMWC. These findings provide ample testimony to demonstrate that blocking the TLR4/NF-κB signalling pathway plays a prominent role in the anti-inflammatory function of LMWC against inflammatory damage during ETEC infection in weaned pigs. Moreover, negative regulatory mechanisms are important to weaken TLR4 signalling and maintain the immunological balance. In this regard, the influences of LMWC on the typical negative regulators of TLR4-mediated inflammatory responses, including Tollip and SIGIRR [38,39], in the ETEC-infected pigs, deserves exploration. Interestingly, we found that LMWC ingestion enhanced the jejunal and ileal mucosal *Tollip* and *SIGIRR* mRNA levels in the ETEC-infected pigs. Therefore, it is probable that the restrained effects of LMWC on the TLR4/NF-κB signalling pathway may be partially related to the up-regulated expression of their negative regulators.

Apoptosis, a mode of cell death, plays a crucial role in the maintenance of intestinal homeostasis [40]. Given the widespread and critical role of apoptosis in enterocyte biology, it is not surprising that dysregulation or excessive apoptosis frequently occurs during pathophysiological disturbances [41]. Indeed, inflammation derived from ETEC infection involves the exacerbation of apoptosis, which is associated with loss of the intestinal barrier integrity [42]. Therefore, we evaluated the effects of LMWC on intestinal epithelial cell apoptosis in ETEC-challenged pigs. As the results showed, LMWC assuaged the jejunal and ileal epithelial cell apoptosis in weaned pigs infected with ETEC. To date, research has indicated that there are two main apoptotic approaches: the intrinsic pathway (mitochondrial pathway) and the extrinsic pathway (death receptor pathway) [43]. Intrinsic apoptosis, which is induced by diverse intracellular stresses, is regulated by Bcl-2 and its relatives, and predominantly leads to caspase-9 activation [44,45]. Extrinsic apoptosis is triggered when death receptors (e.g., Fas and TNFR1) on the plasma membrane, engaged by cognate ligands of the TNF superfamily, recruit caspase-8 through the adaptor protein FADD [46,47]. Both the intrinsic and extrinsic apoptotic pathways converge on the activation of caspase-3, which, in turn, activates the rest of the caspase cascade and culminates in apoptotic cell death [48]. In the current study, we noted that LMWC supplementation decreased the jejunal and ileal mucosal *TNFR1* and *FADD* mRNA levels, and the cleaved caspase-3 and -8 contents, in the ETEC-challenged pigs. Thus, it seems that the TNFR1-mediated apoptosis was restrained in intestinal epithelial cells in LMWC-treated ETEC-challenged pigs, which is another possible reason that LMWC alleviates ETEC-induced intestinal injury.

In summary, LMWC supplementation has beneficial effects in maintaining intestinal integrity in ETEC-infected pigs. These functions of LMWC involve suppression of the intestinal inflammatory responses via the TLR4/NF-κB signalling pathway and inhibition of epithelial cell death via TNFR1-mediated apoptosis.

## 4. Materials and Methods

All animal manipulations were performed according to the Animal Management Rules of the Ministry of Health of the People’s Republic of China and approved by the Animal Ethics Committee of Sichuan Agricultural University (under permit number DKYB20131704, 16 March 2018, Chengdu, China).

### 4.1. Animals and Feeding Management

Twenty-four Duroc × Landrace × Yorkshire pigs (aged 24 days), with a mean body weight (BW) of 6.59 (± 0.12) kg, were individually housed in metabolism cages (1.5 m × 0.7 m × 1.0 m) under appropriate environmental conditions (24–26 °C; 60–70% relative humidity; light cycle of 12 h/day), with free access to feed and water during the 2-week experiment. The composition and nutrient levels of the basal diet met the recommended nutrient requirements of the National Research Council [49], and are shown in Table 1. LMWC, with a molecular weight of 20–30 kDa, degree of deacetylation over 85% and viscosity of 4–10 cP, was kindly provided by Jiaxing Korui Biotech Co., Ltd. (Jiaxing, China). The feed intake of each pig was recorded daily, and the BW of each pig was measured at the start and end of the experiment to calculate the ADG, ADFI and G:F.

### 4.2. Experimental Design

The pigs were randomised to three groups (*n* = 8): (1) non-infected control (CON; pigs received the basal diet and infused with sterilised Luria–Bertani culture); (2) ETEC-infected control (ETEC; pigs received the basal diet and infused with ETEC); and (3) ETEC infection + LMWC supplementation (ETEC + LMWC; pigs received the basal diet supplementation with 100 mg/kg LMWC and infused with ETEC). On day 12, pigs in the challenge groups were orally infused with 100 mL of ETEC suspension (serotype O149:K91:K88ac; China Institute of Veterinary Drugs Control, Beijing, China) at 2.6 × 10^9^ colony-forming units/mL, while pigs in the non-infected control group were orally infused with the equivalent volume of sterilised Luria–Bertani culture [50].

### 4.3. Sample Collection

At 08:00 h on day 15, after overnight fasting, 10-mL blood samples were taken from the anterior vena cava into vacuum tubes. After resting at room temperature for 30 min, the blood samples were centrifuged at 3000× *g*/4 °C for 15 min to harvest serum, and then stored at −20 °C before analysis.

The pigs were subsequently euthanised with an intramuscular sodium pentobarbital injection (200 mg/kg BW) [51]. The abdomen was immediately opened, and intestinal tissue samples were removed and flushed with ice-cold phosphate-buffered saline (PBS). Subsequently, 2-cm-portions of the duodenum, jejunum and ileum were either immersed in PBS for flow cytometry or fixed in 4% paraformaldehyde solution for immunofluorescence and immunohistochemical analyses. In addition, approximately 10-cm-length duodenal, jejunal and ileal segments were opened longitudinally and scratched with a glass slide to collect mucosa samples and then snap-frozen in liquid nitrogen and stored at −80 °C for western blot analysis, cytokine and cleaved caspase content measurements and quantitative real-time polymerase chain reaction (qPCR) analysis.

### 4.4. Serum Parameter Measurements

The commercially available assay kits were used for ELISA measurements of the serum cortisol, d-lactic acid, CRH and LPS concentrations (Jiangsu Jingmei Biotechnology Co., Ltd., Yancheng, China), and determination of the AKP and DAO activities (Nanjing Jiancheng Bioengineering Institute, Nanjing, China), respectively. All determinations were done in triplicate and performed according to the manufacturer’s instructions.

### 4.5. Immunofluorescence Assay

For immunofluorescence assay, the duodenal, jejunal and ileal segments were removed from 4% paraformaldehyde solution, transferred to 30% sucrose in PBS overnight and embedded in O.C.T compound (Sakura Finetek Co., Ltd., Tokyo, Japan) on dry ice. Next, one 5-μm-thick section was cut from each sample using a semi-automatic freezing microtome at −20 °C and mounted on silanised slides, followed by permeabilisation with 0.5% Triton X-100 at room temperature for 10 min. After rinsing three times with PBS, the sections were blocked in 10% normal goat serum-supplemented PBS at room temperature for 30 min and incubated overnight at 4 °C with rabbit anti-occludin antibody (1:100 dilution; Abcam plc., Cambridge, UK). After incubation, the sections were rinsed three times in PBS and then incubated with fluorescein-conjugated goat anti-rabbit antibody (Beijing Zhongshan Golden Bridge Biotechnology Co., Ltd., Beijing, China) at room temperature for 1 h. After rinsing three times in PBS, the sections were counterstained with 4′,6-diamidino-2-phenylindole at room temperature for 10 min, to identify nuclei. After another washing with PBS (performed as described above), the sections were cured with anti-fade reagent (Boster Biological Technology Co., Ltd., Wuhan, China). The occludin immunofluorescence images were captured using an Olympus FV1000 laser scanning confocal microscope (Olympus Corporation, Tokyo, Japan).

### 4.6. Western Blot Analysis

For occludin protein abundance determination, total proteins from duodenal, jejunal and ileal tissues were extracted with RIPA buffer (Beyotime Institute of Biotechnology, Shanghai, China). To assess the nuclear and cytoplasmic NF-κB p65 protein abundances, the nuclear and cytoplasmic fractions from duodenal, jejunal and ileal tissues were extracted with NE-PER^TM^ nuclear and cytoplasmic extraction reagents (Thermo Fisher Scientific, Inc., Waltham, MA, USA). The samples, previously analysed for protein concentration by the bicinchoninic acid method [52], were transferred to 1.5-mL centrifuge tubes, diluted with 4× Laemmli sample buffer (Bio-Rad Laboratories, Inc., Hercules, CA, USA) containing 10% β-mercaptoethanol and denatured at 95 °C for 10 min. Next, samples with equal amounts of total protein (40 μg) were fractioned by 10% sodium dodecyl sulphate–polyacrylamide gel electrophoresis and then transferred onto polyvinylidene fluoride (PVDF) membranes (Merck Millipore Ltd., Tullagreen, Ireland). After blocking with 5% bovine serum albumin in Tris-buffered saline containing 0.1% Tween-20 (TBS/T) at room temperature for 1 h, the PVDF membranes were incubated in the presence of primary antibodies overnight at 4 °C under gentle agitation. The specific primary antibodies included the rabbit anti-occludin antibody (1:1000 dilution; Abcam plc.), mouse anti-NF-κB p65 antibody (1:1000 dilution; Cell Signalling Technology, Inc., Danvers, MA, USA), rabbit anti-lamin B1 antibody (1:1000 dilution; Abcam plc.) and rabbit anti-glyceraldehyde-3-phosphate dehydrogenase (GAPDH) antibody (1:1000 dilution; Cell Signalling Technology, Inc.). Following three rinses with TBS/T, the PVDF membranes were incubated with corresponding secondary antibodies, horseradish peroxidase-linked goat anti-rabbit IgG antibody (1:2000 dilution; Cell Signalling Technology, Inc.) or horseradish peroxidase-linked horse anti-mouse IgG antibody (1:2000 dilution; Cell Signalling Technology, Inc.) at room temperature for 1 h. Following three rinses with TBS/T, the PVDF membranes were treated with Clarity™ Western ECL Substrate (Bio-Rad Laboratories, Inc.), and the protein bands were visualised using a ChemiDoc^TM^ XRS+ Imager System (Bio-Rad Laboratories, Inc.). The protein bands were quantified using Quantity One software (version 3.0; Bio-Rad Laboratories, Inc.), and the results were expressed as the ratio of targeted protein to reference protein.

### 4.7. Intestinal Cytokine and Cleaved Caspase Content Determinations

Intestinal mucosal scrapings (including the duodenal, jejunal and ileal mucosae) were homogenised in ice-cold physiological saline (1:9, *w*/*v*) and then centrifuged at 3000× *g*/4 °C for 15 min. The protein concentration of the resultant supernatant verified before IL-1, IL-6, IL-10, TNF-α, TGF-β, IFN-γ, cleaved caspase-3, -8 and -9 contents were determined, using ELISA kits (Jiangsu Jingmei Biotechnology Co., Ltd.). All operations were performed in triplicate, and the intestinal mucosal cytokine and cleaved caspase contents were normalised to the protein concentration in each sample.

### 4.8. Immunohistochemical Analysis

After fixation, the duodenal, jejunal and ileal tissue samples were dehydrated, embedded in paraffin, sectioned (5 μm) and placed onto silanised slides. Once deparaffinised in xylene and rehydrated in gradient ethanol (decreasing concentrations), the slides were placed in 3% H_2_O_2_ in methanol at room temperature for 10 min, to block endogenous peroxidase activity. The slides were then washed thrice with PBS, placed in 10 mmol/L citrate buffer (pH 6.0) and microwaved to boil (10 min), for antigen retrieval. After washing thrice in PBS, the sections were incubated in 10% normal goat serum for 20 min, to eliminate non-specific antibody binding. Subsequently, mouse anti-mast cell chymase antibody (Abcam plc.) or rabbit anti-mast cell tryptase antibody (PL Laboratories, Inc., British Columbia, Canada) was respectively diluted 1:200 or 1:150 in PBS and incubated overnight at 4 °C. After washing thrice with PBS, the sections were incubated with biotinylated goat anti-rabbit or goat anti-mouse IgG antibodies (Beijing Zhongshan Golden Bridge Biotechnology Co., Ltd.) at 37 °C for 30 min. Next, 3,3′-diaminobenzidine was used as the substrate to visualise the bound antibodies, and the sections were counterstained with haematoxylin and mounted with neutral resin. Finally, the sections were imaged using a Motic BA210 digital microscope (Motic China Group Co., Ltd., Xiamen, China), and the image of each slice in at least five different fields was analysed. The integrated optical density of the mast cell tryptase and chymase were detected using the Image-Pro Plus software (version 6.0; Media Cybernetics, Inc., Rockville, MD, USA) at ×200 magnification, and the mast cell chymase and tryptase expression levels were reflected by the average value of the integrated optical density.

### 4.9. Enterocyte Apoptosis Assessment

Duodenal, jejunal and ileal mucosal layers were isolated and homogenised to form a cell suspension by grinding and filtering. The cells were washed twice with ice-cold PBS and suspended in the PBS to adjust the cell concentration to 1 × 10^6^ cells/mL. Next, 5 μL PE Annexin V and 5 μL 7-aminoactinomycin D were added to a 100-μL-aliquot of cell suspension and blended, followed by incubation at room temperature for 15 min in the dark. Lastly, 400 μL Annexin V binding buffer (1×) was added to the mixture, and the apoptotic cells were detected using a CytoFlex flow cytometer (Beckman Coulter, Inc., Brea, CA, USA) within 1 h.

### 4.10. RNA Isolation, cDNA Synthesis and qPCR

Total RNA was extracted from frozen duodenal, jejunal and ileal mucosae, using RNAiso Plus (Takara Biotechnology Co., Ltd., Dalian, China), according to the manufacturer’s instructions. The total RNA integrity was confirmed by 1% agarose gel electrophoresis, and the concentration and quality were verified by ultraviolet spectrophotometry using a NanoDrop 2000 (Thermo Fisher Scientific, Inc.). Next, 1 μg of total RNA was reverse-transcribed into cDNA using a PrimeScript™ RT reagent kit with gDNA Eraser (Takara Biotechnology Co., Ltd.). The following qPCR conditions were used: 42 °C for 2 min, then 37 °C for 15 min, followed by 85 °C for 5 s.

All qPCR reactions were implemented, in triplicate, on a QuanStudio™ 6 Flex Real-Time PCR System (Applied Biosystems, Foster City, CA, USA) using the TB Green™ Premix Ex Taq™ II (Tli RNaseH Plus; Takara Biotechnology Co., Ltd.). Each reaction included a 10-μL master mix consisting of 5 μL of TB Green Premix Ex Taq II (Tli RNaseH Plus, 2×), 0.4 μL each of forward and reverse primers for target/reference genes (listed in Table 2), 0.2 μL of ROX Reference Dye II (50×), 1 μL of cDNA template and 3 μL of diethylpyrocarbonate-treated water. The thermal cycling parameters were as follows: 1 cycle at 95 °C for 30 s, followed by 40 cycles at 95 °C for 5 s and 60 °C for 34 s. To determine the specificity of amplification, a product melting curve analysis was performed at 95 °C for 15 s, 60 °C for 1 min and 95 °C for 15 s. In addition, six points of 10-fold serial dilutions of cDNA were included in each run to obtain the PCR efficiency by generating a standard curve. The 2^−ΔΔCT^ method was used to calculate the relative mRNA level of all target genes after confirmation that the efficiency values were approximately 100% [53].

### 4.11. Statistical Analysis

All data are expressed as mean ± standard error. Statistical analysis was performed by one-way analysis of variance using the general linear model procedures in SAS 9.0 (SAS Institute, Inc., Cary, NC, USA). The differences among treatments were compared using Tukey’s multiple range tests. Statistical significance was set at *P* < 0.05.

## Figures and Tables

**Table 1:** Ingredients and nutrient composition of the basal diet.

Ingredient	Content (%)
Corn (7.8% crude protein)	29.00
Extruded corn (7.8% crude protein)	24.00
Soybean meal (44.2% crude protein)	11.00
Extruded soybean	10.00
Whey powder (low protein)	7.00
Fish meal (62.5% crude protein)	6.00
Soybean protein concentrate	4.00
Sucrose	3.00
Glucose	2.00
Soybean oil	1.50
Limestone	0.70
Dicalcium phosphate	0.48
l-Lysine·HCl (78%)	0.35
NaCl	0.30
Chloride choline	0.15
dl-Methionine	0.15
l-Threonine (98.5%)	0.10
Tryptophan (98%)	0.02
Vitamin premix *	0.05
Mineral premix ^†^	0.20
Total	100
**Calculated composition**	
Digestible energy (MJ kg^−1^)	14.85
Crude protein	19.60
Calcium	0.85
Total phosphorus	0.57
Available phosphorus	0.46
Lysine	1.39
Methionine	0.48
Methionine + Cysteine	0.75
Threonine	0.84
Tryptophan	0.22

* The vitamin premix provided the following per kg of diets: 6000 IU vitamin (V) A, 3000 IU VD_3_, 24 mg VE, 3 mg VK_3_, 1.5 mg VB_1_, 6 mg VB_2_, 3 mg VB_6_, 0.02 mg VB_12_, 14 mg niacin, 15 mg pantothenic acid, 1.2 mg folic acid and 0.15 mg biotin. ^†^ The mineral premix provided the following per kg of diets: 100 mg Fe, 6 mg Cu, 100 mg Zn, 4 mg Mn, 0.30 mg I and 0.35 mg Se.

**Table 2:** Primer sequences for quantitative real-time polymerase chain reaction.

Gene *	Primer Sequence (5′–3′)	Size (bp)	Accession No.
*TLR4*	Forward: TCAGTTCTCACCTTCCTCCTG	166	NM_001113039.2
	Reverse: GTTCATTCCTCACCCAGTCTTC		
*MyD88*	Forward: GATGGTAGCGGTTGTCTCTGAT	148	NM_001099923.1
	Reverse: GATGCTGGGGAACTCTTTCTTC		
*IRAK1*	Forward: CAAGGCAGGTCAGGTTTCGT	115	XM_003135490.4
	Reverse: TTCGTGGGGCGTGTAGTGT		
*TRAF6*	Forward: CAAGAGAATACCCAGTCGCACA	122	NM_001105286.1
	Reverse: ATCCGAGACAAAGGGGAAGAA		
*Tollip*	Forward: GCAGCAGCAACAGCAGAT	133	NM_001315800.1
	Reverse: GGTCACGCCGTAGTTCTTC		
*SIGIRR*	Forward: ACCTTCACCTGCTCCATCCA	205	NM_001315689.1
	Reverse: TTCCGTCATTCATCTCCACCTC		
*Bcl-2*	Forward: AGCATGCGGCCTCTATTTGA	120	XM_021099593.1
	Reverse: GGCCCGTGGACTTCACTTAT		
*Bax*	Forward: CTGACGGCAACTTCAACTGG	200	XM_003127290.5
	Reverse: CGTCCCAAAGTAGGAGAGGA		
*Fas*	Forward: TGATGCCCAAGTGACTGACC	103	NM_213839.1
	Reverse: GCAGAATTGACCCTCACGAT		
*TNFR1*	Forward: CTGGCATTCTTCCTCTTCGTTG	109	NM_213969.1
	Reverse: CCGGCTCTCCCTCCTTTACA		
*TRADD*	Forward: AGGCGTGCTTGGAGGCT	124	XM_021094047.1
	Reverse: GCGAAGATGAAATTCAAACAGC		
*FADD*	Forward: CTGCGACAACGTGGGGA	101	NM_001031797.1
	Reverse: TCAGGTTTCGGGGATACTTC		
*GAPDH*	Forward: ATGGTGAAGGTCGGAGTGAAC	235	NM_001206359.1
	Reverse: CTCGCTCCTGGAAGATGGT		

* *TLR4*, Toll-like receptor 4; *MyD88*, myeloid differentiation factor 88; *IRAK1*, interleukin-1 receptor-associated kinase 1; *TRAF6*, tumour necrosis factor receptor-associated factor 6; *Tollip*, toll-interacting protein; *SIGIRR*, single immunoglobulin interleukin-1 receptor-related molecule; *Bcl-2*, B-cell lymphoma-2; *Bax*, B-cell lymphoma-2-associated X protein; *TNFR1*, tumour necrosis factor receptor 1; *TRADD*, tumour necrosis factor receptor-associated death domain; *FADD*, Fas-associated death domain; *GAPDH*, glyceraldehyde-3-phosphate dehydrogenase.
